# *HvCEBiP*, a gene homologous to rice chitin receptor *CEBiP*, contributes to basal resistance of barley to *Magnaporthe oryzae*

**DOI:** 10.1186/1471-2229-10-288

**Published:** 2010-12-30

**Authors:** Shigeyuki Tanaka, Akari Ichikawa, Kaori Yamada, Gento Tsuji, Takumi Nishiuchi, Masashi Mori, Hironori Koga, Yoko Nishizawa, Richard O'Connell, Yasuyuki Kubo

**Affiliations:** 1 Laboratory of Plant Pathology, Graduate School of Life and Environmental Sciences, Kyoto Prefectural University, Kyoto 606-8522, Japan; 2 Advanced Science Research Center, Kanazawa University, Ishikawa 920-0934, Japan; 3 Department of Bioproduction Sciences, Ishikawa Prefectural University, Ishikawa 921-8836, Japan; 4 Division of Plant Sciences, National Institute of Agrobiological Sciences, Ibaraki 305-8602, Japan; 5 Department of Plant Microbe Interactions, Max Planck Institute for Plant Breeding Research, Carl von Linné Weg 10, D-50829 Köln, Germany; 6 Department of Organismic Interactions, Max Planck Institute for Terrestrial Microbiology. Karl-von-Frisch-Strasse 35043 Marburg, Germany

## Abstract

**Background:**

Rice CEBiP recognizes chitin oligosaccharides on the fungal cell surface or released into the plant apoplast, leading to the expression of plant disease resistance against fungal infection. However, it has not yet been reported whether CEBiP is actually required for restricting the growth of fungal pathogens. Here we evaluated the involvement of a putative chitin receptor gene in the basal resistance of barley to the *ssd1 *mutant of *Magnaporthe oryzae*, which induces multiple host defense responses.

**Results:**

The *mossd1 *mutant showed attenuated pathogenicity on barley and appressorial penetration was restricted by the formation of callose papillae at attempted entry sites. When conidial suspensions of *mossd1 *mutant were spotted onto the leaves of *HvCEBiP*-silenced plants, small brown necrotic flecks or blast lesions were produced but these lesions did not expand beyond the inoculation site. Wild-type *M. oryzae *also produced slightly more severe symptoms on the leaves of *HvCEBiP*-silenced plants. Cytological observation revealed that these lesions resulted from appressorium-mediated penetration into plant epidermal cells.

**Conclusions:**

These results suggest that *HvCEBiP *is involved in basal resistance against appressorium-mediated infection and that basal resistance might be triggered by the recognition of chitin oligosaccharides derived from *M. oryzae*.

## Background

To resist attack by microbial pathogens, plants have evolved to recognize them, triggering the expression of diverse defense reactions. The currently accepted model is that plants recognize conserved pathogen-associated molecular patterns (PAMPs) through corresponding pattern recognition receptors (PRRs) which in turn trigger plant immune responses [1-3]. The involvement of PRRs in disease resistance against bacterial pathogens is well-documented. For example, the N-terminal amino acid sequence of bacterial flagellin (designated as flg22) can be recognized through the corresponding receptor FLS2 in *Arabidopsis thaliana *[4,5]. In addition, the N-terminal sequence of bacterial translational elongation factor Tu (designated as elf18) can be recognized through the corresponding receptor EFR [6,7].

In contrast to bacterial PAMP receptors, much less is known about the role of fungal PAMP receptors in plants. It is conceivable that oligosaccharides derived from chitin or glucan may function as PAMPs because they are major structural components of fungal cell walls and can induce the expression of several defense-related genes when they are applied to plants [8,9]. The rice plasma membrane glycoprotein CEBiP (Chitin Elicitor Binding Protein) was shown to be an important component for chitin-derived signaling and is thought to be a receptor for fungal PAMPs [10]. CEBiP was identified as a chitin-binding protein from suspension cultured rice cells and contains two LysM (lysin) domains which mediate binding to oligosaccharides. Physiological experiments suggest that CEBiP is required for the production of reactive oxygen species by rice plants in response to treatment with chitin elicitor [10]. It is assumed that CEBiP recognizes chitin oligosaccharides present on the fungal cell surface or released into the plant apoplast, leading to the expression of plant disease resistance against fungal infection. However, it has not yet been reported whether CEBiP is actually required for restricting the growth of fungal pathogens in rice.

*Magnaporthe oryzae *is an ascomycete fungus that causes the devastating blast disease in rice [11]. In the previous report, we have generated *ssd1 *mutants in *M. oryzae *and the cucumber anthracnose fungus *Colletotrichum orbiculare*, in which infection of their respective host plants was restricted by cellular defense responses [12]. Subsequently, by inoculating the *C. orbiculare ssd1 *mutant onto *Nicotiana benthamiana *plants in which defense-related genes were silenced, we evaluated the involvement of those genes in basal defense. These experiments revealed that plants in which genes encoding specific MAPKK (MEK2) and MAPKs (SIPK/WIPK) had been silenced were susceptible to the *ssd1 *mutant, as well as the wild-type strain [13]. Furthermore, we revealed that these MAPKs were activated by fungal cell surface components during infection and that the level of MAPK activation induced by the *ssd1 *mutant was higher than by the wild-type strain, suggesting that MAPK signaling is required for enhanced basal defense and restriction of fungal infection. In addition, use of the *ssd1 *mutant together with gene-silenced plants allowed us to critically evaluate the involvement of specific defense-related genes in basal resistance by assessing whether the *ssd1 *mutant could produce disease lesions on the silenced plants.

In plants, RNA interference (RNAi) is a powerful tool for the evaluation of gene function [14]. For RNAi, it is necessary to generate transgenic plants that express a partial fragment of the target gene, but considerable time is required to obtain seeds from T_1 _transformants. In contrast, virus-induced gene silencing (VIGS) is a simple, rapid method to transiently generate knock-down plants that avoids the need for stable transformation [15]. Although procedures for VIGS are not yet established for rice, there are reports that VIGS is applicable to barley through the use of barley stripe mosaic virus (BSMV) [16,17]. Barley is a susceptible host plant for *M. oryzae*, so that interactions between *M. oryzae *and barley provide a model for the molecular analysis of compatible interactions between monocot plants and fungal pathogens [18].

In this study, we have exploited the barley-*Magnaporthe *pathosystem to evaluate the involvement in basal resistance of genes encoding a putative PAMP receptor, namely *HvCEBiP*, which is homologous to the rice chitin receptor CEBiP. For this, we used the *M. oryzae ssd1 *mutant and BSMV-mediated gene silencing. We present evidence that *HvCEBiP *contributes to basal defense against appressorium-mediated infection by *M. oryzae *in barley.

## Results

### *Magnaporthe oryzae SSD1 *is required for infection of barley

**Figure 1:**
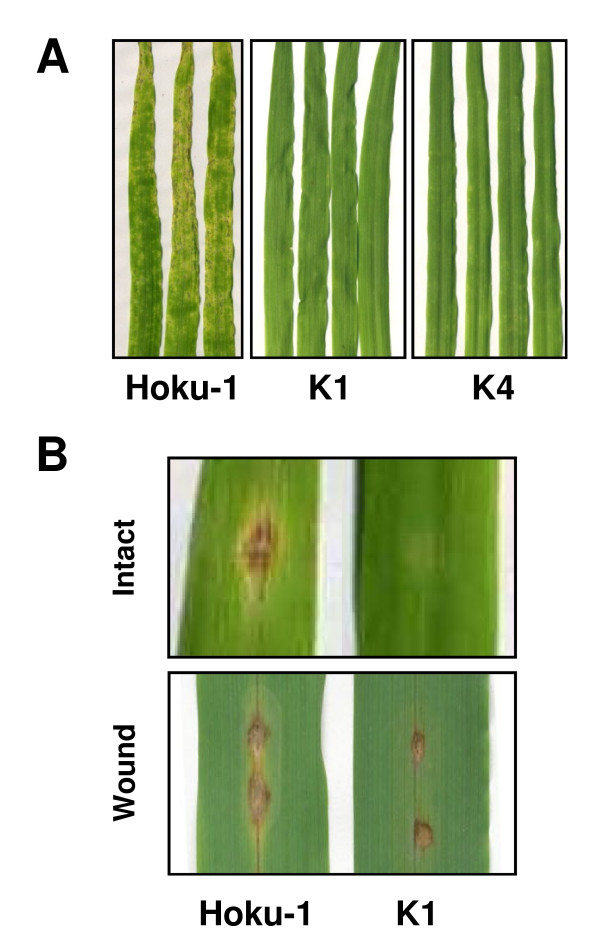
**Pathogenicity of *M. oryzae ssd1 *mutant against barley**. (A) Pathogenicity assay by spray inoculation of the wild-type strain Hoku-1, and *mossd1 *mutants K1 and K4. Conidial suspension (1 × 10^6 ^conidia/ml) was sprayed onto barley leaves and incubated at 24°C. Typical blast lesions were observed on the inoculated leaves with Hoku-1 but not K1 and K4. Photographs were taken 5 days post inoculation. (B) Pathogenicity assay by droplet inoculation of the wild-type Hoku-1 and *mossd1 *mutant K1. Conidial suspensions (1 × 10^5 ^conidia/ml) were spotted onto leaf blades and incubated at 24°C. On intact leaves, severe blast lesions were observed at sites inoculated with Hoku-1, but not K1. On wounded leaves, brown deposition were observed at inoculated sites with both Hoku-1 and K1 but spreading of the lesions only occurred with Hoku-1.

In previous work we showed that the *SSD1 *gene of *M. oryzae *is essential for the successful infection of susceptible rice plants, and that the failure of *mossd1 *mutants to infect was associated with the accumulation of reactive oxygen species (ROS) by host cells [12]. First, we examined whether the *SSD1 *gene is also essential for the infection of barley (*Hordeum vulgare*). When conidial suspensions of the wild-type strain Hoku-1 were inoculated onto leaves, necrotic lesions similar to those of rice blast disease could be observed at 4 days post inoculation (dpi). In contrast, leaves inoculated with the *mossd1 *mutants K1 and K4 did not show visible disease symptoms (Figure 1A). When conidial suspensions were spotted onto intact leaf blades of barley, mutant K1 did not produce any disease symptoms, although the wild-type Hoku-1 forms typical blast lesions at inoculation sites at 4 dpi (Figure 1B). To test whether the K1 mutant retained invasive growth ability, conidial suspensions were spotted onto wound sites on the surface of barley leaves. The mutant produced brown necrotic flecks at wound sites but disease symptoms did not spread further, in contrast to the wild-type Hoku-1 which could form typical blast lesions after infection through wounds (Figure 1B). Overall, the pathogenicity of the *M. oryzae ssd1 *mutants was severely attenuated on barley, producing an infection phenotype similar to that seen previously on rice [12].

**Figure 2:**
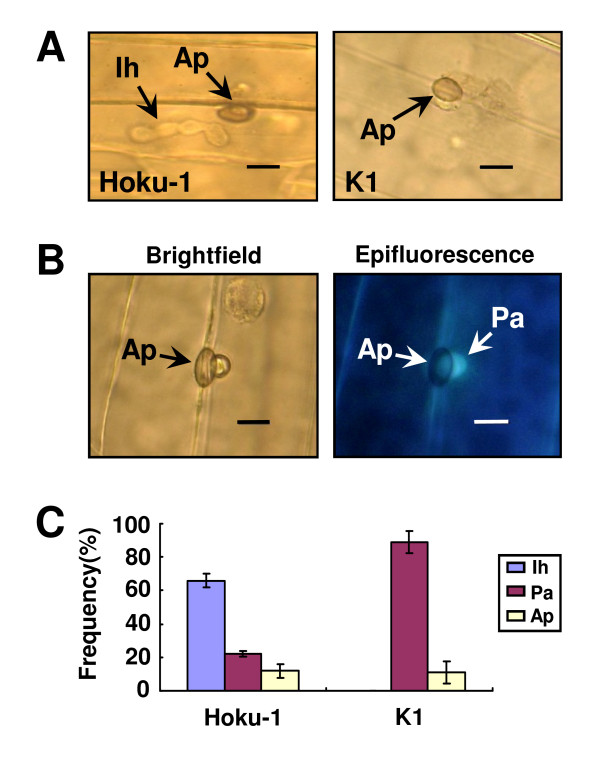
**Cytology of infection of barley leaf tissue by the *M. oryzae ssd1 *mutant**. (A) Infection phenotypes of the wild-type Hoku-1 and *mossd1 *mutant K1. Inoculated leaves at 48 hpi were decolorized and observed with light microscopy. The wild-type strain Hoku-1 formed infection hyphae from appressoria on the plant surface but *mossd1 *mutant K1 did not show infection hyphae inside plant cell. Ap, appressorium; Ih, infection hypha; Bar = 5 μm. (B) Formation of papilla-like structures under appressoria of *ssd1 *mutant K1. At 48 hpi, the decolorized leaves were observed with epi-fluorescence microscopy. Autofluorescent papillae were visible beneath appressoria. Ap, appressorium; Pa, papilla; Bar = 5 μm. (C) Frequency of appressorial penetration and host papilla formation. Leaves sprayed with conidial suspension (1 × 10^6 ^conidia/ml) were observed at 48 hpi. Infection phenotypes were classified as follows; Ih, infection hyphae under appressoria; Pa, papilla under appressoria; Ap, appressoria without papillae or infection hyphae. Appressoria of the wild-type strain Hoku-1 penetrated with high frequency to form infection hyphae, but those of *ssd1 *mutant K1 induced papillae with high frequency.

Microscopic analysis showed that the *mossd1 *mutant formed appressoria on the plant surface indistinguishable from those of the wild-type strain Hoku-1 (Figure 2A). However, while Hoku-1 produced intracellular infection hyphae inside host epidermal cells, mutant K1 had formed no infection hyphae at 48 hpi (Figure 2A). To observe the responses of *H. vulgare *cells to attempted infection by the mutant, inoculated leaves were stained with 3,3'-diaminobenzidine (DAB) to detect H_2_O_2 _accumulation. However, no significant accumulation of H_2_O_2 _was detectable in host cells after inoculation with Hoku-1 or K1 at 48 hpi (data not shown). Next, we attempted to detect the formation of autofluorescent papillae under appressoria using epi-fluoresence microscopy [18]. At sites of attempted penetration by the *mossd1 *mutant, autofluorescent papilla-like structures could be observed beneath approximately 80-90% of mutant appressoria (Figure 2B), and intracellular infection hyphae were only rarely observed inside host cells (Figure 2C). On the other hand, the frequency of papilla formation under appressoria of Hoku-1 was only 20% and infection hyphae developed from 60% of appressoria (Figure 2C). These results suggest that the localized deposition of cell wall material (papillae) at attempted fungal entry sites forms part of the basal defense response of barley epidermal cells to appressorial penetration by *M. oryzae*.

### Virus-induced gene silencing of *HvCEBiP *using barley stripe mosaic virus

**Figure 3:**
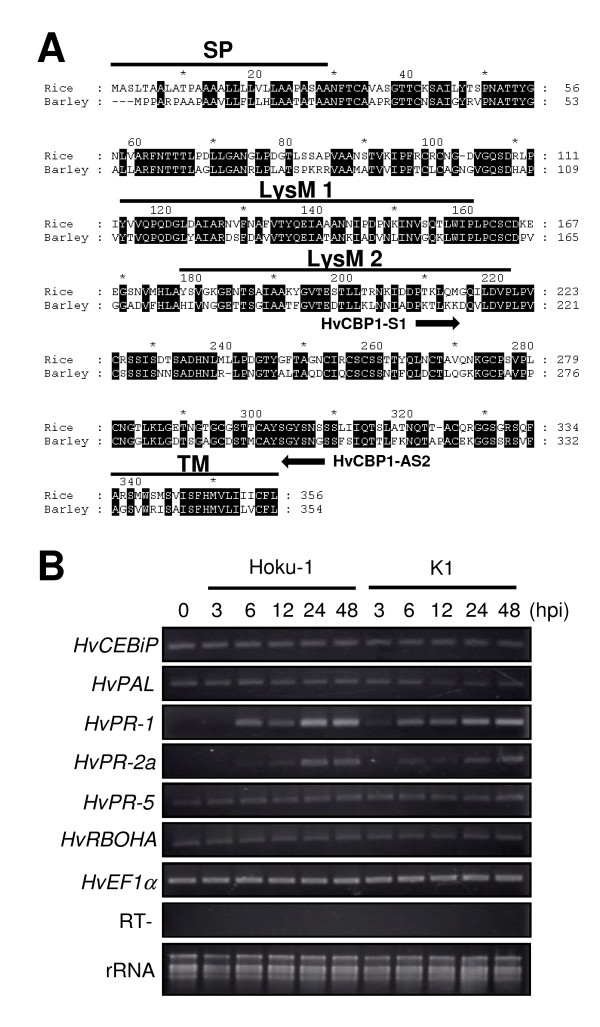
**Sequence and expression profiling of *HvCEBiP***. (A) Alignment of the amino acid sequences between rice CEBiP (Rice) and barley HvCEBiP (Barley). Putative coding sequence of HvCEBiP was aligned with rice CEBiP. Identical amino acids are highlighted with black boxes. SP, signal peptide; LysM 1/LysM 2, LysM motif; TM, transmembrane region. Arrows indicated primer position used for gene silencing of *HvCEBiP*. (B) Expression profiling of *HvCEBiP *and several defense-related genes. Conidial suspensions (1 × 10^5 ^conidia/ml) of the wild-type strain Hoku-1 or *mossd1 *mutant K1 were spotted onto barley leaves and total RNAs were extracted from inoculated tissues at 0 (no inoculation), 3, 6, 12, 24 and 48 hpi for RT-PCR. The expression of *HvCEBiP *was detectable with similar transcript levels at all time points in the leaves inoculated with either Hoku-1 or K1. The expression of *HvPAL*, *HvRBOHA *and *HvPR-5 *was detectable at all time points, but expression of *HvPR-1 *and *HvPR-2a *was induced after inoculation with *M. oryzae*. For checking genomic contamination, PCR of *HvEF1α *was performed using total RNA as template (designated as RT-). Ribosomal RNAs are presented as loading control.

Chitin is major structural component of fungal cell walls and is therefore likely to function as a PAMP [10]. We therefore searched for a gene homologous to the *CEBiP *chitin receptor of rice using a barley EST database (TIGR plant transcript assemblies; http://blast.jcvi.org/euk-blast/plantta_blast.cgi) and found an assembled sequence TA30910_4513 which contains the putative full-length coding sequence. The predicted amino acid sequence showed 66% identity to rice CEBiP. Furthermore, this sequence contained a signal peptide at the N-terminus, and two LysM motifs and a transmembrane region in the C-terminal region, which are all present in rice CEBiP (Figure 3A). Therefore, we consider this gene is very likely to be orthologous to rice CEBiP, and accordingly designated the gene *HvCEBiP*. When we examined the expression of *HvCEBiP *during the course of infection of barley by *M. oryzae *(Figure 3B), transcripts were detectable at all time points (3, 6, 12, 24, 48 hpi), indicating that *HvCEBiP *is likely to be constitutively expressed in barley. In addition, we also examined the expression of selected defense-related genes during infection. Genes homologous to phenylalanine ammonia lyase, respiratory burst oxidase homologue A and pathogenesis-related proteins 1, 2, and 5 were searched from the barley EST database, and designated as *HvPAL*, *HvRBOHA*, *HvPR-1*, *HvPR-2a *and *HvPR-5*, respectively. As shown in Figure 3C, transcripts of *HvPAL*, *HvRBOHA *and *HvPR-5 *could be detected at all time points, suggesting they are constitutively expressed. However, it should be noted that both *PAL *and *PR5 *generally belong to multi-gene families and we cannot exclude that gene members other than those evaluated in this experiment may be inducible by fungal infection. *HvPR-1 *and *HvPR-2a *expression could not be detected at 0 hpi (no inoculation) but was detected from 6 hpi, suggesting the expression of *HvPR-1 *and *HvPR*-*2a *was induced by inoculation with *M. oryzae*. However, there were no major differences in plant defense gene expression induced by the wild type and *mossd1 *mutant K1.

**Figure 4:**
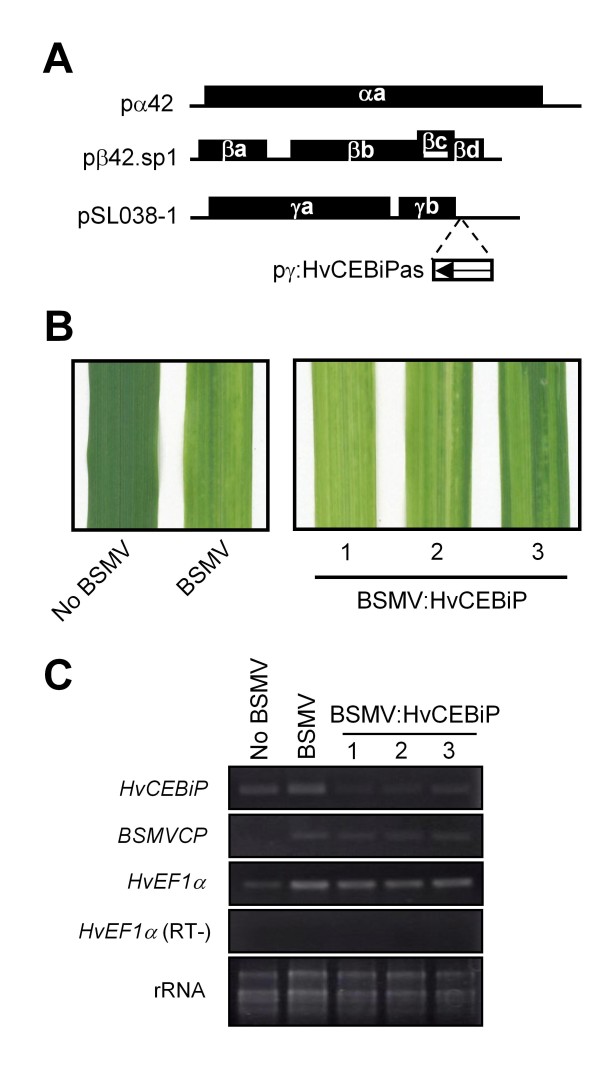
**Evaluation of *HvCEBiP *gene silencing**. (A) The genomic organization of BSMV and corresponding silencing constructs. Genomic RNA of BSMV was transcribed *in vitro *from pα42, pβ42.sp1 and pSL038-1, carrying the α, β and γ genomes, respectively. Genomic RNA of BSMV:HvCEBiP was from pα42, pβ42.sp1 and pγ:HvCEBiPas, which harbours a partial fragment of *HvCEBiP *in the antisense orientation. (B) The third-developed leaves of barley plants at 10 days after inoculation with BSMV genomic RNA onto the first leaves. Stripe mosaic symptoms were observed in the third leaves of BSMV- or BSMV:HvCEBiP-treated plants but not in untreated plants (No BSMV). (C) Evaluation of the silencing effect by RT-PCR. Total RNAs were extracted from the leaves shown in **B **and used for RT-PCR. *BSMVCP *encoding viral coat protein was detectable in BSMV- or BSMV:HvCEBiP-treated plants but not in untreated plant (No BSMV). The expression level of *HvCEBiP *was down-regulated in the third leaves of BSMV:HvCEBiP-treated plants compared to a BSMV-treated plant or untreated plant. For checking genomic contamination, PCR of *HvEF1α *was performed using total RNA as template (RT-). Ribosomal RNAs are presented as loading control.

Next, to evaluate the involvement of *HvCEBiP *in basal resistance of barley, we attempted to perform virus-induced gene silencing (VIGS) using the barley stripe mosaic virus (BSMV) [17]. Before silencing *HvCEBiP*, we first confirmed the efficiency of BSMV-mediated gene silencing in barley by silencing a gene encoding phytoene desaturase (PDS). After BSMV:PDS genomic RNA was inoculated into the first developed leaves of barley plants, a photobleaching phenotype typical of PDS deficiency was visible on the third developed leaves of all inoculated plants, indicating that BSMV-mediated gene silencing of PDS was effective in barley (see Additional file 1: Figure S1). For silencing of *HvCEBiP*, we first amplified a 298 bp partial fragment of *HvCEBiP *from barley leaf cDNA and introduced it into plasmid pSL038-1 which carries the γ genome of BSMV. The resulting construct, in which a fragment of the target gene is introduced in the antisense orientation, was designated as pγ:HvCEBiPas (Figure 4A). The sequence used for silencing *HvCEBiP *did not contain either of the two LysM motifs (Figure 3A). In the EST data base background, we selected unique sequences to HvCEBiP, although without access to the complete barley genome, we could not exclude that there might be other potential CEBiP homologs that are silenced. Next, we attempted to evaluate the silencing effect of *HvCEBiP *by RT-PCR. After inoculation of BSMV:HvCEBiP onto first-developed barley leaves, total RNA was extracted from the third-developed leaves and used for reverse transcription. Typical viral disease symptoms were observed in the third leaves of plants treated with BSMV (control) or BSMV:HvCEBiP genomic RNA (Figure 4B). In these leaves, the expression of both *BSMVCP*, encoding the BSMV coat protein, and *HvEF1α*, encoding barley translational elongation factor, was detectable (Figure 4C). On the other hand, the third leaves of plants treated with BSMV:HvCEBiP showed reduced transcription levels of *HvCEBiP *compared to control plants treated with BSMV (Figure 4C). These results indicate that the transcript level of *HvCEBiP *was down-regulated by BSMV:HvCEBiP-mediated gene silencing in barley.

### *HvCEBiP *contributes to restricting infection by *mossd1 *mutants

**Figure 5:**
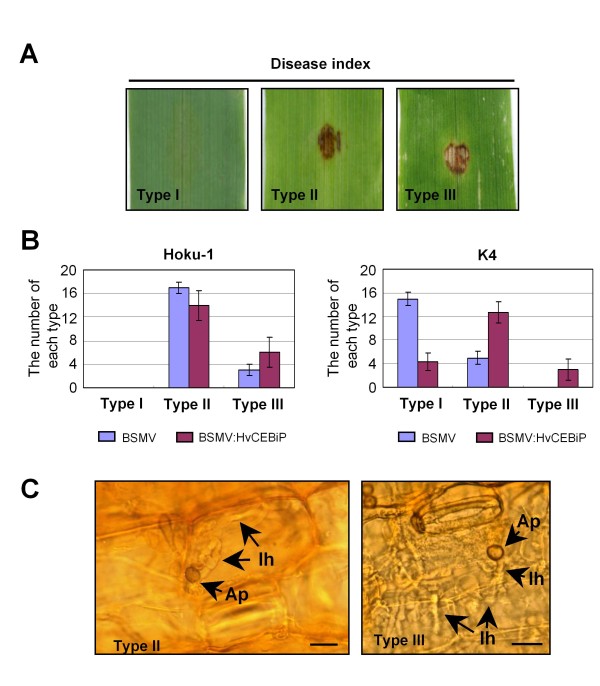
**Pathogenicity of *M. oryzae ssd1 *mutant on the third leaves of BSMV:HvCEBiP-treated barley plants**. (A) Disease symptom index on barley leaves inoculated with *M. oryzae*: Type I, no visible disease symptoms; Type II, brown necrotic flecks; Type III, severe blast lesion with less brown necrotic flecks. (B) Quantification of disease symptoms at 7 dpi according to the disease index shown in (A). Conidial suspensions (1 × 10^5 ^conidia/ml) of the wild-type strain Hoku-1 or *mossd1 *mutant K4 were spotted onto the third leaves of BSMV- or BSMV:HvCEBiP-treated plants. Mutant K4 produced a greater frequency of Type II and Type III infections on BSMV:HvCEBiP-treated plants than on BSMV-treated plants. on BSMV:HvCEBiP-treated plants, the wild-type Hoku-1 also produced slightly more severe symptoms (type III) than on BSMV-treated plants. Twenty droplet inoculations were performed in each experiment with three biological replicates. Data represent mean numbers of inoculation sites and error bars = 1 standard deviation. (C) Cytology of appressorium-mediated infection by *ssd1 *mutant K4 on leaves of BSMV:HvCEBiP-treated plants. In Type II lesions, infection hyphae emerging from appressoria were observed inside only one epidermal cell, without further hyphal growth into adjacent cells. Formation of infection hyphae was associated with death of the penetrated cell. In Type III lesionsssss, infection hyphae developed further, colonizing neighboring cells, without visible host cell death. Ap, appressorium; Ih, infection hypha; Bar = 10 μm.

To examine whether *HvCEBiP *is involved in the basal resistance of barley to *Magnaporthe*, we inoculated the *mossd1 *mutant K4 onto the third-developed leaves of barley plants after inoculation of BSMV:HvCEBiP onto the first-developed leaves. To quantify the severity of disease symptoms produced by the *mossd1 *mutant, we classified disease symptoms as follows; Type I, no visible symptoms; Type II, brown necrotic flecks; Type III, blast lesions without brown necrotic flecks (Figure 5A). On the leaves of BSMV-treated plants, most symptoms produced by *mossd1 *mutant K4 were classified as Type I (Figure 5B), whereas on leaves of BSMV:HvCEBiP-treated plants Type II symptoms were produced at approximately half of the sites inoculated with K4 (Figure 5B). This tendency was confirmed in three independent experiments. When the wild-type strain Hoku-1 was inoculated onto leaves of BSMV:HvCEBiP-treated plants, the frequency of Type III symptoms was slightly but consistently higher compared to the control plant, although these effects were not statistically significant (Figure 5B). When conidial suspensions were inoculated onto wound sites on the leaves of BSMV:HvCEBiP-treated plants, there was no significant difference in disease symptoms produced by Hoku-1 and K4 (data not shown), suggesting that the silencing of *HvCEBiP *does not affect invasive growth ability through wounds. Taken together, these results suggest that *HvCEBiP *is involved in basal defense responses of susceptible barley plants to appressorial penetration by *M. oryzae*.

To determine whether the *mossd1 *mutant was able to develop infection hyphae and colonize barley tissues, we observed leaf inoculation sites in BSMV:HvCEBiP-treated plants at 96 hpi. At sites showing brown necrotic flecks (Type II symptom), appressoria were present on the leaf surface, and infection hyphae developed from appressoria inside the initially infected epidermal cell, which appeared to undergo a cell death reaction (Figure 5C). However, when we observed inoculation sites at 7 dpi, fungal hyphae had not colonized the neighboring host cells and hyphae were entirely confined to the first infected cell (data not shown). These observations suggest that *mossd1 *mutant appressoria could penetrate into *HvCEBiP*-silenced plants but subsequent growth of the infection hyphae became restricted by host defense responses. However, at the few inoculation sites showing severe lesions (Type III), infection hyphae were seen to develop from appressoria without visible host cell death (Figure 5C). Taken together, these results suggest that *HvCEBiP *contributes to host defense responses expressed after invasion of epidermal cells by *M. oryzae *infection hyphae.

**Figure 6:**
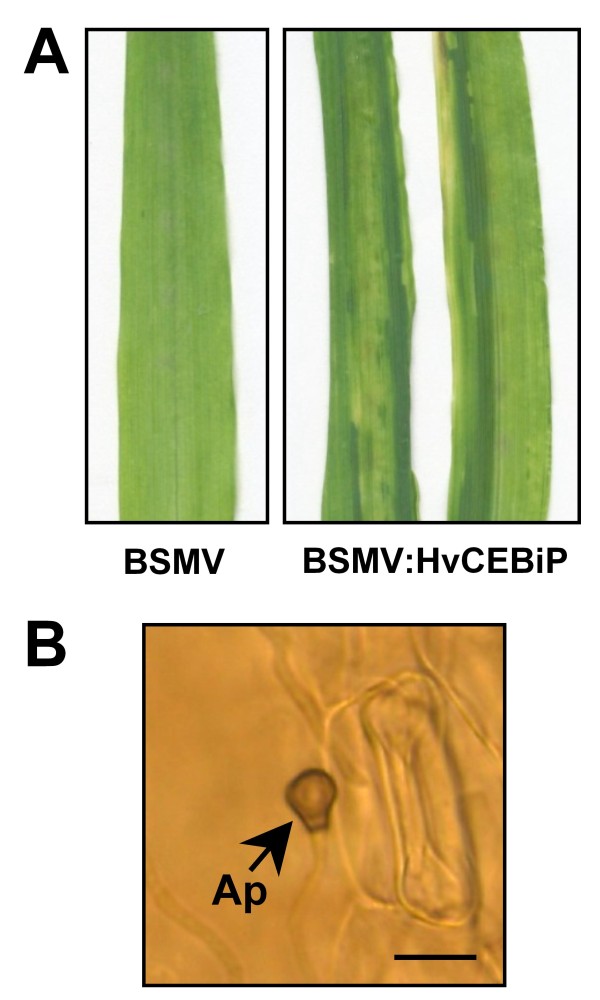
**Pathogenicity of nonadapted pathogen *Colletotrichum graminicola *on barley**. (A) photographs of the inoculated leaves of BSMV:HvCEBiP-treated plants. Droplets of conidial suspension of *C. graminicola *were applied onto the leaves and photographs were taken at 96 hpi. (B) Microscopy showed that *C. graminicola *could form appressoria on BSMV:HvCEBiP-treated plants but could not penetrate epidermal cells to form infection hyphae. Bar = 10 μm.

To evaluate whether *HvCEBiP *is also involved in non-host resistance, we inoculated conidia of the non-adapted maize anthracnose pathogen *C. graminicola *onto the third leaves of BSMV:HvCEBiP-treated plants. Although *C. graminicola *formed appressoria on the leaves of both BSMV- and BSMV:HvCEBiP-treated plants, intracellular infection hyphae were not observed, and no disease symptoms were produced (Figure 6). This suggests that *HvCEBiP *does not play a critical role in resistance to non-adapted pathogens such as *C. graminicola*.

**Figure 7:**
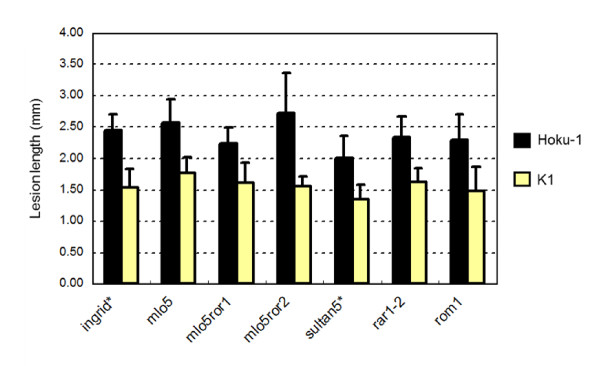
**Pathogenicity test of the wild-type Hoku-1 and *mossd1 *mutant K1 on a range of barley mutants affected in various defense-related genes**. Droplets of conidial suspension were applied onto leaves of genetic mutants of *mlo5*, *Ror1*, *Ror2*, *Rar1 *and *Rom1*. Ingrid is the wild-type cultivar for *mlo5*, *ror1 *and *ror2 *mutants. Sultan5 is the wild-type cultivar for *rar1 *and *rom1*.

Next, we evaluated the possible role in basal defense of selected barley genes required for penetration resistance and *R*-gene mediated resistance to the powdery mildew fungus, *Blumeria graminis *f. sp. *hordei*. For this, we used barley mutant lines deficient in *Ror1 *and *Ror2 *(required for *mlo*-specified resistance) [19,20], *Rar1 *(required for *Mla12 *resistance) [21] and *Rom1 *(restoration of *Mla12*-specified resistance) [22]. After inoculating conidial suspension of *mossd1 *mutant K4 onto leaves of these barley mutants, no significant differences in symptom severity were observed compared to the respective wild-type barley cultivars (Figure 7). It therefore appears that none of these genes are involved in restricting infection by the *mossd1 *mutant.

### Expression profiling of defense-related genes in *HvCEBiP*-silenced plants

**Figure 8:**
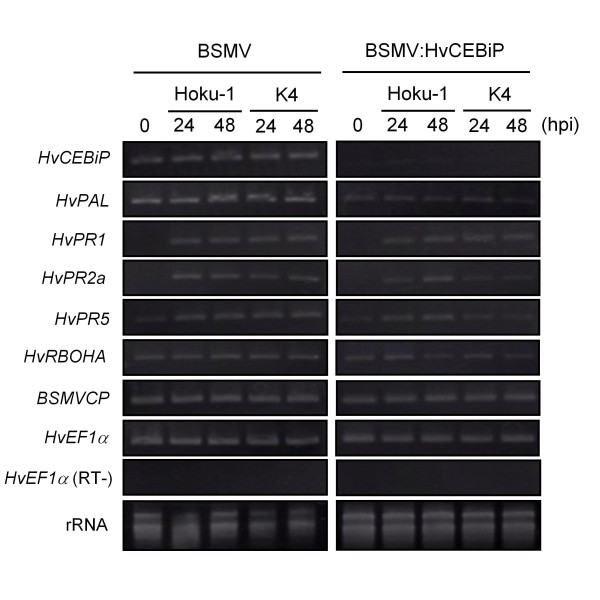
**Expression profiling of defense-related genes in leaves of BSMV:HvCEBiP-treated plants**. Total RNAs were extracted from the leaves of BSMV- or BSMV:HvCEBiP-treated plants inoculated with *M. oryzae *wild-type strain Hoku-1 or *mossd1 *mutant K4 at 0 (no inoculation), 24 and 48 hpi. The expression of *HvCEBiP *was strongly down-regulated in BSMV:HvCEBiP-treated plants compared to BSMV-treated plants. The expression of *HvPAL, HvPR-2a *and *HvPR-5 *was also down-regulated in BSMV:HvCEBiP-treated plants compared to BSMV-treated plants. In contrast, the expression levels of *HvPR-1 *and *HvRBOHA *in BSMV:HvCEBiP-treated plants were similar to those in BSMV-treated plants.

To identify plant defense-related genes that may be regulated by HvCEBiP-mediated signaling, we evaluated the expression patterns of selected barley defense genes in the leaves of BSMV:HvCEBiP-treated plants (Figure 8). Total RNAs were extracted at 0 h (no inoculation), 24 h and 48 h after inoculation of the wild-type Hoku-1 or *mossd1 *mutant K4 onto leaves of BSMV- or BSMV:HvCEBiP-treated plants. The expression of *HvEF1α *and *BSMVCP *was detected at all time points. In contrast, the expression of *HvCEBiP *was clearly down-regulated in BSMV:HvCEBiP-treated plants, confirming that *HvCEBiP *had been silenced. The expression of *HvPAL, HvPR-2a *and *HvPR-5 *also appeared to be down-regulated in BSMV:HvCEBiP-treated plants compared to BSMV-treated plants. However, the expression levels of *HvPR-1 *and *HvRBOHA *in BSMV:HvCEBiP-treated plants were similar to those in BSMV-treated plants. These results suggest that the expression of *HvPAL*, *HvPR-2a *and *HvPR-5 *might be regulated by *HvCEBiP *signaling.

## Discussion

### Barley expresses two layers of basal defense in response to infection by *Magnaporthe oryzae*

In our previous study, we generated an *ssd1 *mutant of *M. oryzae*, in which the infection of rice plants was restricted by a defense response involving death of the initially infected epidermal cell [12]. This cell death reaction expressed by rice in response to compatible isolates of *M. oryzae *has been termed 'whole-plant specific resistance' (WPSR), and is independent of *R*-gene mediated resistance in rice [23,24]. In the present study, infection assays revealed that the *mossd1 *mutant also showed attenuated pathogenicity on barley. However, the host defense responses expressed in barley to appressorial penetration by the *mossd1 *mutant took the form of papilla deposition at attempted fungal entry sites rather than host cell death. The phenomenon of papilla formation during *M. oryzae *infection of barley has also been reported by other authors [18]. In rice, papilla-like wall appositions were also observed beneath appressoria of *M. oryzae*, although these appeared small and thin with electron microscopy [25]. Therefore, the formation of papillae appears to be a general form of basal defense against attempted appressorial penetration by *M. oryzae *in barley. However, the efficiency of papillae in restricting appressorial penetration seems to be weak because the wild-type strain could successfully penetrate into plant cells with high frequency, as shown in Figure 2C. Apart from papilla formation, a localized cell death reaction was also observed in the initially penetrated host cells in which infection hyphae had developed. This cell death reaction was observed in the leaves of BSMV:HvCEBiP-treated barley plants after infection by both the *ssd1 *mutant and the wild-type strain of *M. oryzae*. The cell death reaction was associated with inhibition of fungal growth because infection hyphae had not developed beyond the first infected epidermal, even after 7 days. The barley cell death reaction resembles WPSR in rice [23] and conceivably it represents a basal defense response triggered after successful penetration by *M. oryzae *appressoria. It therefore appears that barley deploys two distinct layers of basal defenses against appressorium-mediated infection by *M. oryzae*, namely papilla formation and localized cell death. Two similar layers of plant defense were also shown to operate during non-host resistance of *Arabidopsis *to powdery mildew fungi [26].

### *HvCEBiP *is involved in basal resistance to appressorial penetration by *M. oryzae*

In our recent work, we used the *C. orbiculare ssd1 *mutant to show that a specific MAPK pathway in *N. benthamiana *plays a critical role in host basal defense but genes required for *R*-gene mediated resistance (*RAR1*, *SGT1 *and *HSP90*) do not [13]. Here, we used the *M. oryzae ssd1 *mutant to examine the role in basal defense of genes required for penetration resistance and *R*-gene mediated resistance. *Ror1 *and *Ror2 *were identified as genes required for *mlo*-specific resistance against the barley powdery mildew fungus *Blumeria graminis *f. sp. *hordei *and *Ror2 *shows functional homology to syntaxin AtSYP121 in *Arabidopsis *[27]. *Rar1 *was originally shown to be required for race-specific resistance triggered by resistance gene *Mla12 *against *B. graminis *f. sp. *hordei *expressing the avirulence gene *AvrMla12 *[28,29]. *Rom1 *was identified as a restoration of *Mla12*-specified resistance (*rom1*) mutant that restores disease resistance to *B. graminis *f. sp. *hordei *carrying the avirulence gene *AvrMla12 *[22]. However, infectivity of the *mossd1 *mutant was not significantly enhanced on any of these barley mutants compared to wild-type plants, suggesting that genes required for *R*-gene mediated resistance do not play a role in basal defense against *M. oryzae*, consistent with findings from the *C. orbiculare*-*N. benthamiana *interaction [13].

**Figure 9:**
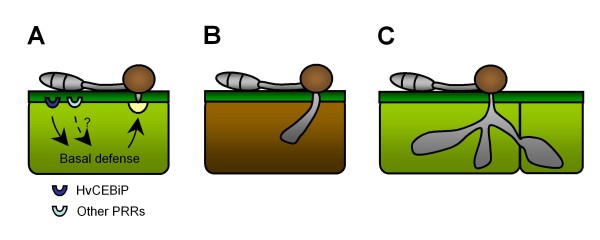
**Working model for the involvement of *HvCEBiP *to dual layers basal defense in *M. oryzae*-barley interaction**. (A) When an *M. oryzae *appressorium attempts to penetrate a barley epidermal cell, host basal defenses based on the formation of papillae are induced by the recognition of *M. oryzae *by HvCEBiP or other pattern recognition receptors (PRRs). However, this basal defense is insufficient to inhibit appressorial penetration by the wild-type strain, which successfully establishes infection hyphae inside living host cells. In contrast, appressorial penetration by the *mossd1 *mutant is effectively restricted by the formation of papillae at attempted entry sites. (B) When infection hyphae of the *mossd1 *mutant successfully invade barley epidermal cells in *HvCEBiP*-silenced plants, a second layer of basal defense, associated with death of the initially infected cell, leads to restriction of hyphal development. This localized cell death also occurs in leaves inoculated with the wild-type strain, and may therefore be a general defense response to infection by *M. oryzae*. (C) When the wild-type strain successfully develops infection hyphae inside the initially infected cell without cell death reaction, the wild-type attempts the further infection to neighboring cells by development of infection hyphae.

In contrast to mutations in these barley genes, the knock-down of HvCEBiP did enhance infection by the *mossd1 *mutant. Thus, on BSMV:HvCEBiP-treated plants mutant K4 produced more severe (Type II) symptoms, i.e. brown necrotic flecks, compared to BMSV-treated control plants (Figure 5B). The silencing of *HvCEBiP *also increased the frequency of successful appressorial penetration by the *mossd1 *mutant. However, the formation of infection hyphae inside penetrated epidermal cells appeared to trigger localized host cell death, resulting in brown necrotic symptoms. These results suggest that *HvCEBiP *is involved in basal defense against appressorial penetration by *M oryzae*. In contrast to the *mossd1 *mutant, infectivity of the wild-type strain was not significantly enhanced on *HvCEBiP*-silenced plants but there was a slight increase in symptom severity. This suggests that although *HvCEBiP *contributes to basal defense in barley, the level of its contribution may be low, so that with the highly pathogenic wild-type strain differences in symptoms between non-silenced and *HvCEBiP*-silenced plants were hard to distinguish. One plausible explanation of these findings is that basal defense against appressorial penetration involves multiple PAMP receptors and signaling pathways, of which signaling *via *HvCEBiP is only one. A working model for the contribution of *HvCEBiP *to the dual-layered basal defense responses of barley to *M. oryzae *is presented in Figure 9.

In addition to the increased frequency of brown necrotic fleck symptoms induced by the *mossd1 *mutant on BSMV:HvCEBiP-treated plants, a few inoculation sites also showed formation of severe blast lesions (Type III symptom) as shown in Figure 5A. Lesion formation was not associated with localized cell death reactions and infection hyphae developed extensively, colonizing many host cells. This suggests that in some cases the *mossd1 *mutant was able to infect *HvCEBiP*-silenced plants without triggering cell death-associated defense responses. This raises the possibility that *HvCEBiP *might be involved in mediating the localized cell death response of barley epidermal cells to invasion by *M. oryzae *infection hyphae. Thus, *HvCEBiP *might contribute not only to papilla-based defenses but also to the hypersensitive cell death response to cell invasion. *HvCEBiP *does not appear to play a central role in nonhost resistance because the non-adapted pathogen *C. graminicola *produced no symptoms on silenced plants. In contrast, the LysM domain receptor kinase CERK1 was reported to contribute weakly to the resistance of *Arabidopsis thaliana *against the incompatible pathogen *Alternaria brassicicola *[30].

### Is *HvCEBiP *a specific receptor for components of the *mossd1 *mutant?

In the interaction between cucumber anthracnose pathogen *C. orbiculare *and *N. benthamiana*, we reported previously that the altered fungal cell wall composition conferred by *ssd1 *gene disruption triggers plant basal resistance through the activation of a specific plant MAPK cascade [13]. We hypothesized that activation of the MAPK pathway might result from recognition of fungal PAMP(s) by corresponding plant receptor protein(s). In this study, we attempted to determine whether HvCEBiP is a specific receptor for PAMPs expressed uniquely by the *mossd1 *mutant, in which case pathogenicity of the wild-type strain should not be affected by the silencing of *HvCEBiP*. However, the wild-type strain Hoku-1 showed a slight increase in pathogenicity on *HvCEBiP*-silenced plants, suggesting that HvCEBiP is a receptor for component(s) shared by both the wild-type *M. oryzae *and *mossd1 *mutant.

Rice CEBiP is a receptor-like protein containing two LysM domains, which was originally identified in enzymes that degrade the bacterial cell wall component peptidoglycan [31]. Recent biochemical analysis showed that the LysM domain can also mediate binding to chitin oligosaccharides [32]. The genome of *Arabidopsis *contains five LysM domain-containing receptor-like kinases [33], among which CERK1 (At3g21630) was identified as a receptor-like protein required for chitin signaling in *Arabidopsis *[30]. Although the function of the other LysM domain-containing receptor-like kinases is unknown, it is tempting to speculate that plants possess multiple receptor proteins for the perception of particular classes of pathogen-derived oligosaccharides. It is likely that other PAMP receptors, in addition to HvCEBiP, are conserved in barley and contribute to basal resistance to *M. oryzae*.

## Conclusions

Rice CEBiP recognizes chitin oligosaccharides derived from fungal cells leading to the expression of plant disease resistance against fungal infection. We evaluated the involvement of putative chitin receptor gene *HvCEBiP *in barley basal resistance using the *mossd1 *mutant of *Magnaporthe oryzae*, which enhances host basal defense responses. The *mossd1 *mutant showed attenuated pathogenicity on barley and appressorial penetration was restricted by the formation of papillae at attempted entry sites. On *HvCEBiP*-silenced plants, the mutant produced small brown necrotic flecks or blast lesions accompanied by appressorium-mediated penetration into plant epidermal cells. Wild-type *M. oryzae *also produced slightly more severe symptoms on the leaves of *HvCEBiP*-silenced plants. These results indicated that *HvCEBiP *is involved in basal resistance against appressorium-mediated infection and that basal resistance could be triggered by the recognition of chitin oligosaccharides derived from *M. oryzae*.

## Methods

### Plant growth conditions and fungal strains

*Hordeum vulgare *wild-type cultivars Fiber-snow, Ingrid and Sultan5, and genetic mutants *mlo5*, *mlo5ror1*, *mlo5ror2*, *rar1 *and *rom1 *were grown in a controlled environment chamber (16 h photoperiod, 24°C). *Magnaporthe oryzae *Hoku-1 was used as the wild-type strain in this study. The *mossd1 *mutants K1 and K4 were generated as reported previously [12]. These fungal cultures were maintained at 24°C on oatmeal agar medium (6.0 g powder oatmeal, 1.25 g agar per 100 ml distilled water) under continuous light. *Colletotrichum graminicola *isolate MAFF236902 was described previously [13].

### Pathogen inoculation and cytological assays

To induce conidiation, two week-old cultures of *M. oryzae *were washed with sterile water to remove aerial hyphae and then incubated for a further 3 days. For inoculation, conidial suspension was sprayed (5 ml; 1 × 10^6 ^conidia/ml) or spotted (10 μl; 1 × 10^5 ^conidia/ml) onto the third leaves of *H. vulgare *and incubated in a humid plastic box at 24°C. For evaluation of invasive growth ability, the surface of barley leaves was scratched with a sterile plastic pipette tip and droplets of conidial suspensions were placed directly onto the wound sites. Cytological observations and the detection of papillae were performed as follows. Inoculated leaves were cut to 1 cm × 1 cm size and decolorized with a 3:1 mixture of ethanol:chloroform and mounted under a coverslip in lactophenol solution. Autofluorescent papillae formed beneath appressoria were visualized by epifluoresence. The accumulation of H_2_O_2 _in host cells was detected by staining with 3,3'-diaminobenzidine [13].

### RT-PCR

Total RNA was extracted from barley leaves using TRIzol Reagent (Invitrogen) following the manufacturer's protocol. RT-PCR was performed using ReverTra Dash RT-PCR kit (Toyobo) following the manufacturer's protocol. The primers used for RT-PCR are listed in Additional file 1: Table S1. The sequence data of *HvPAL*, *HvPR-1*, *HvPR-2a*, *HvPR-5*, *HvRBOHA *and *HvEF1α *can be found in GeneBank with accession numbers Z49147, Z21494, AY612193, AF355455, AJ871131 and Z50789, respectively.

### Vector construction

A 298 bp partial fragment of *HvCEBiP *was amplified by primer pairs HvCBP1-S1 (5'-CCAAAGACCCTCAAGAAGGA-3') and HvCBP1-AS1 (5'-AGCCGTTGGAATAACCACTG-3') from cDNA of *H. vulgare *and subcloned into the pGEM-T easy vector (Promega). The resulting construct was digested by *Not*I and a fragment containing the amplified sequence of *HvCEBiP *was introduced into the *Not*I site of pSL038-1 in the antisense orientation. This construct was designated as pγ:HvCEBiPas.

### Virus-induced gene silencing

BSMV genomic RNAs were transcribed *in vitro *as previously described with some modifications [17]. The reaction was performed at 37°C for 60 min in 50 μl of reaction buffer containing 1 μg of linearized plasmids, 1 μl of T7 RNA polymerase (Takara), 10 μl of 50 mM DTT, 6 μl of 10 mM NTPs (rATP, rCTP, rUTP), 0.4 μl of 10 mM rGTP and 5 μl of 5 mM m^7^G(ppp)G RNA cap structure analog (New England Biolabs). After the reaction, 1.62 μl of 10 mM rGTP and 1 μl of T7 RNA polymerase were added to the reaction mixture, and further incubated at 37°C for 60 min. Transcribed α, β, γ genomic RNAs were mixed in a 1:1:1 ratio with 20 μl FES and inoculated onto the first-developed leaves of *H. vulgare *plants with gentle rubbing. The third-developed leaves were used for evaluating fungal infections.

## Authors' contributions

ST designed the experiments, performed the gene silencing study and wrote the manuscript. AI performed the sample preparations and vector construction. KY performed the inoculation assay for barley mutant lines. GT participated in experimental procedures for PCR analysis. HK participated in cytological analysis of barley infection assay. MM participated in barley gene silencing and data analysis, TN participated in barley infection assay and data analysis. NY participated in experimental procedures concerning CEBiP and data analysis. RO supervised the study and critically revised the manuscript. YK conceived and directed the whole study, and participated in the writing of the manuscript. All authors read and approved the final manuscript.

## Supplementary Material

Additional file 1 **Figure S1**. Efficiency of BSMV-mediated gene silencing in barley. (A) photobleaching by gene silencing of phytoene desaturase (PDS) in barley. BSMV:PDS was inoculated onto the first developed leaf (1). After 10 days, photobleacing was observed in the third developed leaf (3). (B) close-up photograph of third- and fourth- developed leaves shown in A. (C) photobleaching phenotypes in five individual plants treated with BSMV:PDS. Third leaves of all five plants showed photobleaching. **Table S1**. Primers used for RT-PCR. Click here for file
