# Supplementary material for: HvCEBiP, a gene homologous to rice chitin receptor CEBiP, contributes to basal resistance of barley to Magnaporthe oryzae
Source: BMC Plant Biol. 2010 Dec 30;10:288. doi: 10.1186/1471-2229-10-288 (PMC3020183; doi:10.1186/1471-2229-10-288)
Supplement: Additional file 1 — Figure S1. Efficiency of BSMV-mediated gene silencing in barley. (A) photobleaching by gene silencing of phytoene desaturase (PDS) in barley. BSMV:PDS was inoculated onto the first developed leaf (1). After 10 days, photobleacing was observed in the third developed leaf (3). (B) close-up photograph of third- and fourth- developed leaves shown in A. (C) photobleaching phenotypes in five individual plants treated with BSMV:PDS. Third leaves of all five plants showed photobleaching. Table S1. Primers used for RT-PCR. [file 1471-2229-10-288-S1.PDF]

Figure S1

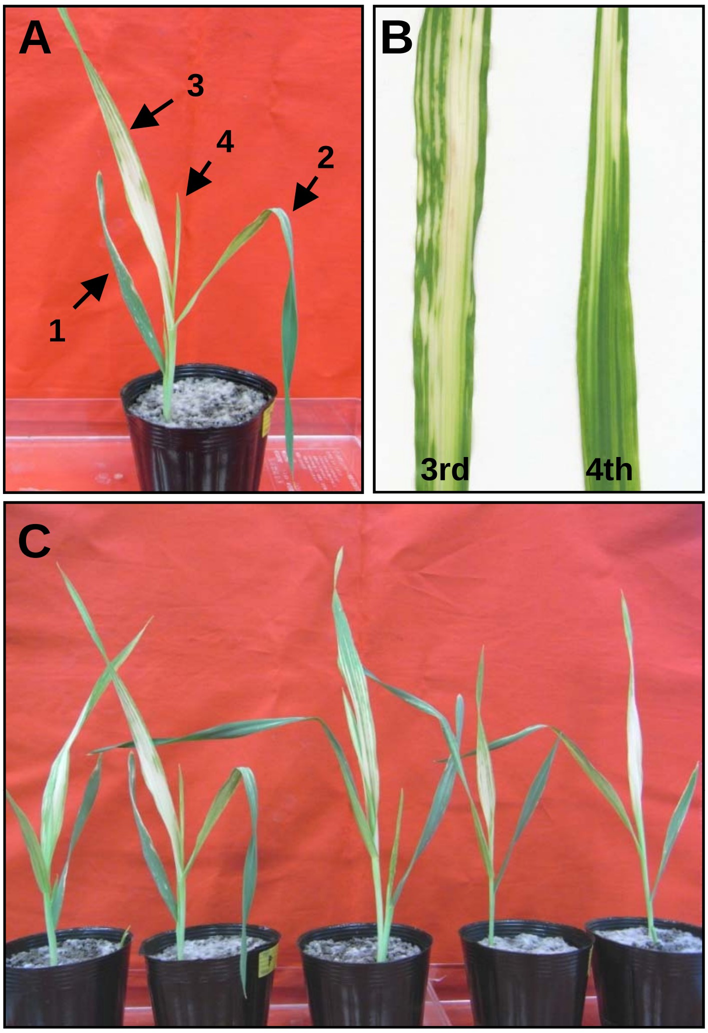

Figure S1. Efficiency of BSMV-mediated gene silencing in barley. **A,** photobleaching by gene silencing of phytoene desaturase (PDS) in barley. BSMV:PDS was inoculated onto the first developed leaf (1). After 10 days, photobleacing was observed in the third developed leaf (3). **B,** close-up photograph of third- and fourth- developed leaves shown in **A**. **C,** photobleaching phenotypes in five individual plants treated with BSMV:PDS. Third leaves of all five plants showed photobleaching.

Table S1

Table S1. Primers used for RT-PCR

Primer name	Sequence (5'- 3')	Amplification target
HvCBP1-S1	CCAAAGACCCTCAAGAAGGA	*HvCEBiP*
HvCBP1-AS2	GCTGGTGCTGTCTGGTTTTT	
HvPAL-S1	ACGGACGGACACGTTCTC	*HvPAL*
HvPAL-AS1	TATCTGTCCAGGGTGGTGCT	
HvPR1-S1	CCAAGCTAGCCATCTTGCTC	*HvPR-1*
HvPR1-AS1	TGGAGCCGTAGTCGTAGTCC	
HvPR2a-S1	CAGGGCCTCAACATCAAGTA	*HvPR-2a*
HvPR2a-AS1	AGGTTCTGGTTGTACGTCTGC	
HvPR5-S1	GGAGCTTCTCCATCACGAAC	*HvPR-5*
HvPR5-AS1	GCTGCAAGCTTTGGTCTTG	
HvRBOHA-S1	TGGCACTCATCCTCCTACCT	*HvRBOHA*
HvRBOHA-AS1	CCCAACAGGAACTGCAAGAT	
BSMVCP-S1	TTTAGCGTTGCTTCCTCTCG	*BSMV CP*
BSMVCP-AS1	GGGGGAGATGGGTTAGTTGT	
HvEF1a-S1	CACCATCGATATTGCCCTCT	*HvEF1*α
HvEF1a-AS1	CAGTCAAGGTTGGTGGACCT	
